# Comparison of Three Immunoassays for the Detection of Myositis Specific Antibodies

**DOI:** 10.3389/fimmu.2019.00848

**Published:** 2019-04-30

**Authors:** Michael Mahler, Zoe Betteridge, Chelsea Bentow, Michaelin Richards, Andrea Seaman, Hector Chinoy, Neil McHugh

**Affiliations:** ^1^Inova Diagnostics, Inc., San Diego, CA, United States; ^2^Department of Pharmacy and Pharmacology, University of Bath, Bath, United Kingdom; ^3^Rheumatology Department, Salford Royal NHS Foundation Trust, Manchester Academic Health Science Centre, Salford, United Kingdom; ^4^NIHR Manchester Biomedical Research Centre, Manchester University NHS Foundation Trust, The University of Manchester, Manchester, United Kingdom

**Keywords:** myositis, autoantibodies, diagnosis, polymyosits, dermatomyositis, immunoassay

## Abstract

**Objectives:** Standardization of myositis specific antibody (MSA) detection is of high importance because these antibodies are relevant for diagnosis and stratification of patients with idiopathic inflammatory myositis (IIM) and have the potential to be used in classification criteria. Many laboratories rely on immunoprecipitation (IP) for the detection of MSA but this approach is compromised by logistic, standardization, and regulatory challenges. Therefore, reliable alternatives to IP are mandatory. Here we aimed to compare three methods for the detection of MSA.

**Methods:** Our study initiated from a cohort of 1,619 IIM patients (BIRD/University of Bath serology service and UKMyoNet cohorts) and resulted in 157 unique serum samples enriched for higher prevalence of MSA characterized by the laboratory's routine methods, IP and line immunoassay (LIA: Euroimmun). All samples were tested using a novel fully automated particle-based multi-analyte technology (PMAT, Inova Diagnostics, research use only). Analyses included antibodies to PL-7, PL-12, SRP, NXP2, Mi-2, SAE, EJ, MDA5, TIF1γ, SRP, NXP2.

**Results:** Overall high agreements were observed between novel methods (LIA and PMAT) and IP (Cohen's *kappa* 0.46–0.96) for the detection of MSA. Lowest level of agreement was found for EJ and highest for SAE.

**Conclusion:** The data hold promise for advancements in standardization of MSA assays as well as for the potential inclusion of MSA in future classification criteria.

## Introduction

Myositis specific (MSA) and myositis associated antibodies (MAA) have been used as an aid in the diagnosis of idiopathic inflammatory myopathies (IIM) for decades (1). Since many of the MSA (e.g., anti-synthetase antibodies), partly depending on the screening dilution, are accompanied by limited sensitivity of the indirect immunofluorescence (IIF) test, this method has limited utility as a screening test for suspected myositis (2–6). Especially during the past 10–15 years, many novel and clinically relevant MSA have been identified (1) which can help in the diagnosis and stratification of IIM. Since the publication of updated classification criteria for IIM (7, 8), a debate has been triggered about the exclusion of MSA (except anti-Jo-1 antibodies), which was eventually explained by the lack of standardization of autoantibody assays and missing data derived from large multi-centric studies (9, 10). Shortly afterwards, an alternative classification approach was proposed that leverages both clinical and autoantibody data (11). The aim of the present study was to compare the results from three methods for the detection of MSA, including radiolabeled protein immunoprecipitation (IP), a commonly used line immunoassay (LIA), and a newly developed particle-based multi-analyte technology (PMAT) using a large cohort of IIM patients.

## Methods

From an original cohort of 1,619 IIM patients (Bath Institute for Rheumatic Diseases (BIRD)/University of Bath serology service and UKMyoNet cohorts) (12), a total of 157 patients were selected based on MSA characterized by the laboratory's routine methods, IP [as described in (13)] and line immunoassay (LIA: Euroimmun not FDA approved; OJ, EJ, PL-12, PL-7, SRP, Jo-1, Ro52, PM-75, PM-100, Ku, SAE1, NXP2, MDA5, TIF1γ, Mi-2β, Mi-2α). Demographic data was available for the majority of the patients: Median age (of 118 patients with age data available) was 53.0 years (range 4.0–83.0 years), 101 were female, 43 males (of 144 patients with gender data available). The clinical phenotypes of patients were dermatomyositis (DM, *n* = 76), polymyositis (PM, *n* = 31), myositis of unknown subtype (UM, *n* = 15), overlap syndromes (*n* = 11), juvenile DM (JDM, *n* = 8), anti-synthetase syndrome (ASS, *n* = 7), clinically amyopathic DM (CADM, *n* = 5), and immune-mediated necrotizing myopathy (IMNM, *n* = 4). Written consent to participate and to provide biological samples was obtained from all subjects according to the Declaration of Helsinki, under the local ethical committee regulations of each participating center. The study of autoantibodies in myositis patients was reviewed and approved by the North West Research Multi-center Research Ethics Committee 98/8/86. LIA results were interpreted semi-quantitatively by estimating intensities (0–3) according to instructions for use and samples >0 were defined as positive. All samples were tested using a novel PMAT system (Inova Diagnostics, research use only; PL-7, PL-12, SRP, NXP2, Mi-2, SAE, EJ, OJ, MDA5, TIF1γ and HMGCR). For the PMAT, antigens were coupled to paramagnetic particles that carry unique signatures and incubated with diluted patient samples (final sample dilution of 1:200). After 9.5 min incubation at 37°C, particles were washed and incubated 9.5 min at 37°C with anti-human IgG conjugated to phycoeryhtrin (PE). Finally, after another washing cycle, particles were analyzed through digital imaging technology. The cut-off values were previously established using IIM patients (*n* > 250) as well as healthy and disease controls (*n* = 840) using receiver operating characteristic (ROC) analysis. Best combination of sensitivity and specificity was selected. Precision of the novel PMAT system was assessed by testing samples in triplicate in three independent runs over 3 days. Coefficient of variation was expressed in percent. Antibodies to antigens only contained in the LIA and anti-OJ and anti-HMGCR antibodies were not analyzed due to the lack of positive samples or lack of IP data.

## Results

**Figure 1:**
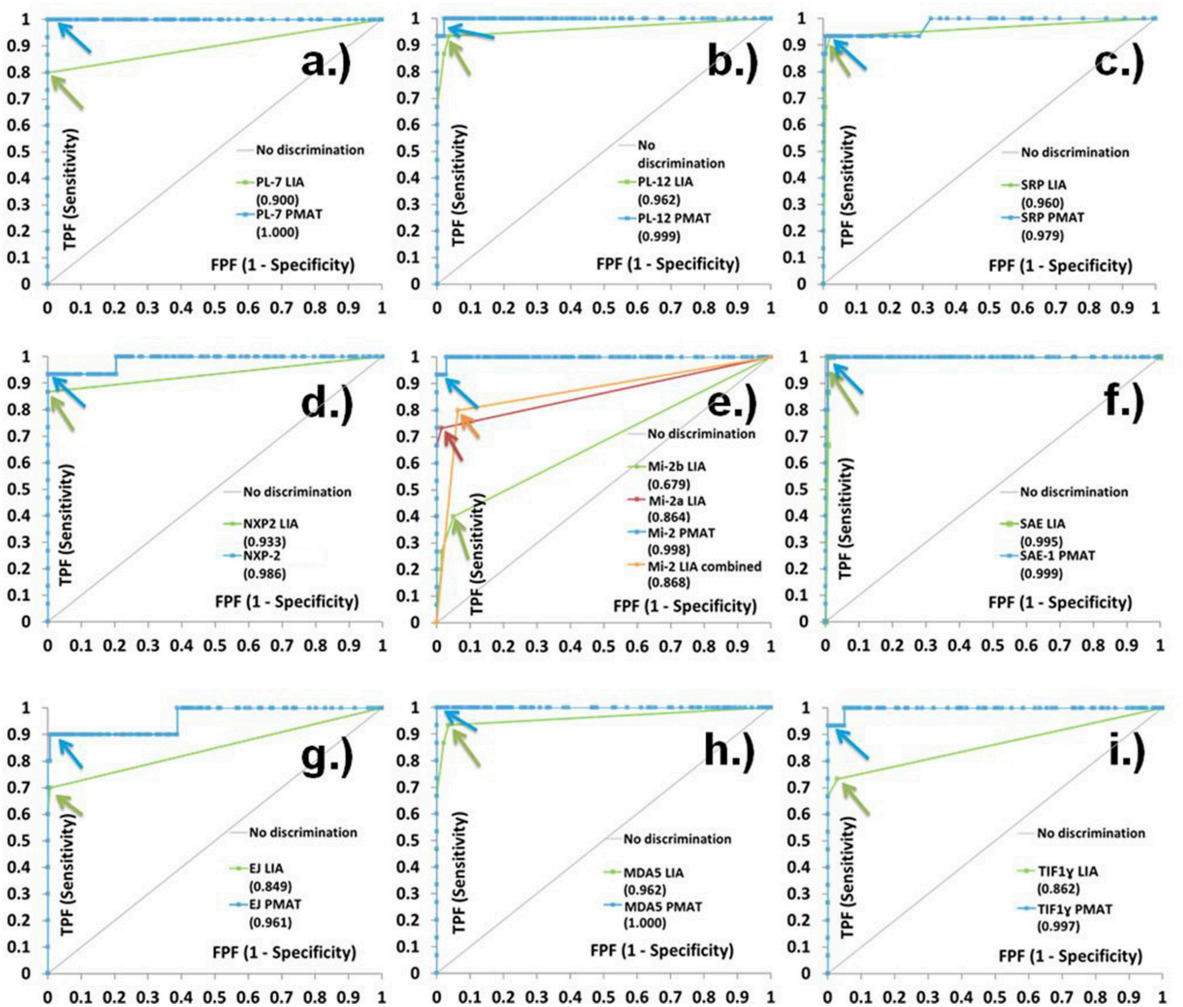
Receiver operating characteristic (ROC) curve analysis of line immunoassay (LIA) and particle-based multi-analyte technology (PMAT) against immunoprecipitation (IP) as binary classifier. Very high level of discrimination between IP positive and negative samples was observed for PMAT system for **(a)** PL-7, **(b)** PL-12, **(c)** SRP, **(d)** NXP2, **(e)** Mi-2, **(f)** SAE, **(g)** EJ, **(h)** MDA5, and **(i)** TIF1γ. LIA results are expressed as grading values (0 = negative, 1–3 = positive according to instructions for use).

When comparing the three assays IP, LIA and PMAT, the comparison showed varying qualitative agreement between the different methods (Cohen's *kappa* 0.46–0.96, see Table 1). Most significant differences among the methods were found for anti-PL-7, anti-Mi-2, anti-EJ, and anti-TIF1γ antibodies. When the results obtained by IP were used as reference (binary classifier) for ROC curve analysis, good discrimination and high area under the curve (AUC) values were found for the PMAT (AUC ≥ 0.82) and for most of the LIA analytes (except Mi-2, AUC = 0.68). For all analytes, the AUC values for PMAT were higher compared to LIA (see Figure 1). The precision study on PMAT demonstrated high consistency with CV% ranging from 1.8 to 5.0% with an average of 3.4%. When the results obtained with the different methods were deciphered in light of the clinical phenotype, IP and PMAT demonstrated agreement with known IIM subsets (see Supplement Table).

**Table 1:** Method comparison of particle-based multi-analyte technology (PMAT) vs. immunoprecipitation (IP) and line immunoassay (LIA).

**Analyte (*n* = IP positives)**	**PMAT vs. IP**		**LIA vs. IP**		**PMAT vs. LIA**	
	**NPA/PPA/TPA**	***Kappa* (95% CI)**	**NPA/PPA/TPA**	***Kappa* (95% CI)**	**NPA/PPA/TPA**	***Kappa* (95% CI)**
PL-7 (*n* = 15)	98.6/100.0/98.7	0.93 (0.83–1.00)	100.0/80.0/98.1	0.88 (0.74–1.00)	96.6/100.0/96.8	0.81 (0.65–0.97)
PL-12 (*n* = 15)	99.3/93.3/98.7	0.93 (0.82–1.00)	96.5/93.3/96.2	0.80 (0.65–0.96)	99.3/73.7/96.2	0.80 (0.65–0.96)
SRP (*n* = 15)	99.3/93.3/98.7	0.93 (0.82–1.00)	99.4/93.3/94.3	0.73 (0.56–0.89)	99.3/63.6/94.3	0.73 (0.56–0.89)
NXP2 (*n* = 15)	98.6/93.3/98.1	0.89 (0.77–1.00)	100.0/86.7/98.7	0.92 (0.81–1.00)	97.9/100.0/98.1	0.89 (0.76–1.00)
Mi-2 (*n* = 15)	100.0/93.3/99.4	0.96 (0.89–1.00)	93.7/80.0/92.4	0.62 (0.43–0.82)	97.8/52.4/91.7	0.58 (0.38–0.79)
SAE (*n* = 15)	99.3/100.0/99.4	0.96 (0.89–1.00)	97.9/100.0/98.1	0.90 (0.79–1.00)	100.0/88.9/98.7	0.93 (0.84–1.00)
EJ (*n* = 10)	95.2/90.0/94.9	0.67 (0.45–0.88)	99.3/70.0/97.5	0.76 (0.54–0.99)	93.3/75.0/92.4	0.46 (0.21–0.72)
MDA5 (*n* = 15)	97.2/100.0/97.5	0.87 (0.74–0.99)	96.5/93.3/96.2	0.80 (0.65–0.96)	97.1/78.9/94.9	0.76 (0.60–0.92)
TIF1γ (*n* = 15)	97.2/93.3/96.8	0.83 (0.69–0.98)	97.2/73.3/94.9	0.71 (0.51–0.90)	95.1/73.3/92.4	0.63 (0.43–0.83)

*NPA, negative percent agreement; PPA, positive percent agreement; TPA, total percent agreement; PMAT, particle-based multi-analyte technology; LIA, line immunoassay; IP, immunoprecipitation; SRP, signal recognition particle; TIF1γ, transcriptional intermediary factor 1 gamma; MDA5, Melanoma differentiation-associated protein 5; NXP2, nuclear matrix protein 2; SAE, small ubiquitin-like modifier activating enzyme*.

## Discussion

Careful evaluation of autoantibody assays for the detection of MSA and MAA is of utmost importance since some of these antibodies are included or being considered for IIM classification criteria (1, 8–10, 14, 15). Although only anti-Jo-1 antibodies have been included in the recent EULAR/ACR classification criteria for IIM, it was acknowledged that several other MSA also carry clinical value. In addition, several autoantibodies showed relevance for a novel approach to classify IIM (11). Consequently, the markers are not only relevant as an aid in the diagnosis, but also in the stratification to specific disease subsets (1, 11, 15). Since most of the clinical associations of MSA and MAA have been established using IP, it is important to also compare newer technologies, such as LIA and PMAT to IP (10). At present, besides IP, mostly LIA and dot blot (DB) assays are routinely used for the detection of MSA, which are convenient tools for the simultaneous detection of various antibodies, but are also accompanied by some limitations including the lack of true quality controls (14), lack of sensitivity for some analytes and subjectivity in interpretation (16). To address the subjectivity of LIA and DB, automated scanning systems have been developed and introduced (16, 17) that allow for a ‘semi-quantitative’ assessment and thus for the estimation of antibody levels (titers).

Several studies have evaluated LIAs for the detection of MSA (17–21), but only a few compared the results to IP. Of relevance, two recent studies comparing LIA and IP demonstrated different levels of agreement for several MSA (19, 22). Whilst IP may not be correct in all instances, it is commonly regarded as the “Gold Standard” technique for IIM autoantibody detection making comparative data invaluable (23). Along those lines, there is no standardized protocol of IP for the detection of autoantibodies.

One of the main challenges for the evaluation of MSA assays is the low prevalence and incidence of IIM and the low prevalence of the individual markers within IIM cohorts, especially the autoantibodies directed to the tRNA synthetases (24). Therefore, most of the conducted comparison studies only included < 5 positive samples for some of the analytes (17–19). In contrast, our study started with a large cohort of 1,619 individual IIM patients which resulted in at least ten positive cases for each of the analytes.

In the present study, we compare results obtained from three methods for the detection of MSA including IP, LIA and a newly developed PMAT. All three methods used significant different technologies and assay protocols. IP is based on the concept of antibody-antigen complexes forming in solution and the precipitated proteins visualized following polyacrylamide gel separation and autoradiography. This approach is able to detect direct and indirect antibody-antigen interactions. LIA are based on membranes (often nitrocellulose) as the solid phase which is able to passively absorb proteins. The detection is based on colorimetric precipitation on the membrane that can be (semi)-quantified. The PMAT system is based on paramagnetic particles with unique signatures with covalently coupled antigens. Read-out of the results is based on digital image analysis. All incubations are carried out under controlled temperature 37°C. Despite the technological differences, overall, we observed mostly high levels of agreement among all the methods. When comparing the *kappa* agreement of PMAT and LIA with IP, for 7/9 analytes PMAT demonstrated higher *kappa* agreement. For the remaining two analytes (NXP2 and EJ), when considering the ROC curves, the cut-off of the PMAT might be too low for EJ resulting in lower negative percent agreement and therefore in a lower *kappa* value. The most pronounced differences between PMAT and LIA were found for anti-SRP, anti-Mi-2, anti-EJ, and anti-TIF1γ antibodies. Underlying reasons for differences might include different antigen sources, epitope exposure, and/or assay conditions. In contrast, SAE showed high level of agreement among all methods which is in line with a previous study (20). In summary, the agreement among the methods was relatively high, especially when compared to other autoantibody assays that are part of classification criteria and are daily used to aid in the diagnosis of autoimmune diseases. As an example, anti-dsDNA antibodies are a classification criteria marker and used in the diagnosis of systemic lupus erythematosus, but the correlation among methods is relatively limited (25). When the results derived from the individual methods were compared with the well-established IIM phenotypes, PMAT and IP showed close correlation. Further studies on unselected patient cohorts are needed to further study the clinical performance for IIM.

The lack of analyte specific calibrators and controls might represent a serious concern. Consequently, reproducibility studies (intra and interassay as well as lot-to-lot) are required to exclude inter-manufacturer variability that may be derived from limited precision and reproducibility. Ideally, those studies should include sufficient samples around the cut-off and follow Clinical and Laboratory Standards Institute (CLSI) guidelines (https://clsi.org/). Another aspect toward better standardization of MSA assays is the access to proficiency testing initiatives as performed for many diagnostic tests. All those needs depend on the availability of control material. Close collaboration between research networks (12), patient groups and kit manufacturers is required to supply serum samples for calibration and quality control. Since it can be challenging to obtain large volume bulk samples, pooling of patient samples or the generation of human monoclonal antibodies could provide viable alternatives (26, 27).

## Ethics Statement

Study was performed using leftover patient sample and study was approved by Bath Institute for Rheumatic Diseases (BIRD)/University of Bath serology service and UKMyoNet cohorts.

## Author Contributions

MM, ZB, and NM established study design. HC contributed data for immunoprecipitation. CB, MR, and AS provided data on the particle-based multi-analyte technology system.

### Conflict of Interest Statement

MM, CB, MR, and AS are employees of Inova Diagnostics (no stocks or shares). The remaining authors declare that the research was conducted in the absence of any commercial or financial relationships that could be construed as a potential conflict of interest.
