# Supplementary material for: Comparison of Three Immunoassays for the Detection of Myositis Specific Antibodies
Source: Front Immunol. 2019 Apr 30;10:848. doi: 10.3389/fimmu.2019.00848 (PMC6503053; doi:10.3389/fimmu.2019.00848)
Supplement: Supplementary file 1 [file Data_Sheet_1.docx]

Supplement table Distribution of autoantibody reactivity among different subforms of myositis derived from particle-based multi-analyte technology (PMAT) versus immunoprecipitation (IP) and line immunoassay (LIA).

| **Subtype** | **Mi-2**  **IP**  **LIA**  **PMAT** | **TIF1**γ  **IP**  **LIA**  **PMAT** | **NXP-2**  **IP**  **LIA**  **PMAT** | **SAE**  **IP**  **LIA**  **PMAT** | **MDA5**  **IP**  **LIA**  **PMAT** | **PL-7**  **IP**  **LIA**  **PMAT** | **PL-12**  **IP**  **LIA**  **PMAT** | **EJ**  **IP**  **LIA**  **PMAT** | **SRP**  **IP**  **LIA**  **PMAT** |
| --- | --- | --- | --- | --- | --- | --- | --- | --- | --- |
| **Alternative name** | **NuRD** | **anti-p155/p140** | **MJ, MORC3** | **SUMO1** | **CADM140** | **Threonyl-tRNA synthetase** | **Alanyl-tRNA synthetase** | **Glycyl-tRNA synthetase** | **Signal recognition particle** |
| **Main clinical association from literature** | **DM** | **DM** | **DM** | **DM** | **CADM** | **ASS** | **ASS** | **ASS** | **IMNM** |
| **DM (n=76)** | 19.7%  18.4%  18.4% | 13.2%  11.8%  18.4% | 15.8%  13.2%  14.5% | 17.1  19.7%  18.4% | 11.8%  11.8%  17.1% | 7.9%  5.3%  9.2% | 5.3%  5.3%  5.3% | 2.6%  3.9%  5.3% | 0.0%  3.9%  1.3% |
| **PM (n=31)** | 0.0%  6.5%  0.0% | 0.0%  6.5%  0.0% | 3.2%  3.2%  6.5% | 0.0%  3.2%  0.0% | 3.2%  6.5%  3.2% | 9.7%  9.7%  9.7% | 9.7%  9.7%  9.7% | 6.5%  6.5%  9.7% | 32.3%  35.5%  32.3% |
| **UM (n=15)** | 0.0%  6.7%  0.0% | 0.0%  6.7%  0.0% | 0.0%  0.0%  6.7% | 0.0%  0.0%  0.0% | 13.3%  20.0%  13.3% | 13.3%  6.7%  20.0% | 6.7%  26.7%  13.3% | 26.7%  13.3%  33.3% | 20.0%  33.3%  20.0% |
| **OM (n=11)** | 0.0%  0.0%  0.0% | 0.0%  0.0%  0.0% | 9.1%  9.1%  9.1% | 0.0%  0.0%  0.0% | 0.0%  0.0%  0.0% | 18.2%  18.2%  18.2% | 18.2%  18.2%  18.2% | 0.0%  0.0%  0.0% | 18.2%  18.2%  9.1% |
| **JDM (n=8)** | 0.0%  25.0%  0.0% | 62.5%  37.5%  50.0% | 12.5%  12.5%  12.5% | 0.0%  0.0%  0.0% | 12.5%  12.5%  12.5% | 0.0%  0.0%  0.0% | 12.5%  12.5%  12.5% | 0.0%  0.0%  12.5% | 0.0%  0.0%  0.0% |
| **ASS (n=7)** | 0.0%  0.0%  0.0% | 0.0%  0.0%  0.0% | 0.0%  0.0%  0.0% | 0.0%  0.0%  0.0% | 0.0%  28.6%  0.0% | 14.3%  14.3%  14.3% | 57.1%  57.1%  42.9% | 28.6%  14.3%  42.9% | 0.0%  0.0%  0.0% |
| **CADM (n=5)** | 0.0%  20.0% 0.0% | 0.0%  0.0%  0.0% | 0.0%  0.0%  0.0% | 40.0%  40.0%  40.0% | 40.0%  40.0%  40.0% | 20.0%  20.0%  20.0% | 0.0%  20.0%  0.0% | 0.0%  0.0%  0.0% | 0.0%  20.0%  0.0% |
| **IMNM (n=4)** | 0.0%  25.0%  0.0% | 0.0%  0.0%  0.0% | 0.0%  0.0%  0.0% | 0.0%  0.0%  0.0% | 0.0%  0.0%  0.0% | 0.0%  0.0%  0.0% | 0.0%  0.0%  0.0% | 0.0%  0.0%  0.0% | 0.0%  0.0%  0.0% |
| **Grand Total** | 9.6%  13.4%  8.9% | 9.6%  9.6%  11.5% | 9.6%  8.3%  10.2% | 9.6%  11.5%  10.2% | 9.6%  12.1%  12.1% | 9.6%  7.6%  10.8% | 9.6%  12.1%  9.6% | 6.4%  5.1%  10.2% | 0.0%  14.0%  9.6% |

DM=dermatomyositis; PM=polymyositis; UM=undifferentiated myositis, OM=overlap myositis; ASS=anti-synthetase syndrome; CADM= clinically amyopathic dermatomyositis; IMNM=immune mediated necrotizing myositis; SRP=signal recognition particle; TIFγ+transcriptional intermediary factor 1 gamma; MDA5=Melanoma differentiation-associated protein 5; NXP2=nuclear matrix protein 2; NuRD=Nucleosome Remodeling Deacetylase; SAE=small ubiquitin-like modifier activating enzyme; SUMO= small ubiquitin-like modifier
